# Supplementary material for: LLM-PBC: Logic Learning Machine-Based Explainable Rules Accurately Stratify the Genetic Risk of Primary Biliary Cholangitis
Source: J Pers Med. 2022 Sep 26;12(10):1587. doi: 10.3390/jpm12101587 (PMC9604872; doi:10.3390/jpm12101587)

SUPPLEMENTARY MATERIAL

LLM-PBC: Logic Learning Machine-based explainable rules accurately stratify the genetic risk of Primary Biliary Cholangitis

Table of Contents

COLLABORATORS 2
SUPPLEMENTARY METHODS 4
SUPPLEMENTARY TABLE LEGEND 6
SUPPLEMENTARY TABLES 8
Supplementary Table 1 8
Supplementary Table 2 9
Supplementary Table 3 10
Supplementary Table 4 15
Supplementary Table 5 22
Supplementary Table 6 23
Supplementary Table 7 24
SUPPLEMENTARY FIGURE LEGEND 25
SUPPLEMENTARY FIGURES 25
Supplementary Figure 1 25

COLLABORATORS

Italian PBC Genetics Study Group
Andrea Affronti[6], Maurizia Brunetto[7], Barbara Coco[7], Giancarlo Spinzi[8], Gianfranco Elia[9], Carlo Ferrari[9], Ana Lleo[4,5], Luigi Muratori[10], Paolo Muratori[10], Piero Portincasa[11], Agostino Colli[12], Savino Bruno[13], Guido Colloredo[14], Francesco Azzaroli[15], Pietro Andreone[16], MariaConsiglia Bragazzi[17], Domenico Alvaro[18], Vincenzo Cardinale[18], Nora Cazzagon[19], Annarosa Floreani[19,] Cristina Rigamonti[20], Floriano Rosina[21], Antonio Ciaccio[1,2], Miki Scaravaglio[1,2], Daphne D'Amato[1,2], Federica Malinverno[1,2], Eleonora Binatti[1,2], Sara Massironi[1,2], Vincenzo Ronca[1,2], Donatella Barisani[1,2], Pietro Lampertico[22], Francesca Donato[22], Stefano Fagiuoli[23], Piero L. Almasio[24], Edoardo Giannini[25], Carmela Cursaro[26], Massimo Colombo[27], Luca Valenti[28], Luca Miele[27], Angelo Andriulli[28], Grazia A. Niro[28], Ignazio Grattagliano[29], Lorenzo Morini[30], Giovanni Casella[31], Maria Vinci[32], Pier Maria Battezzati[33], Andrea Crosignani[33], Massimo Zuin[33], Alberto Mattalia[34], Vincenza Calvaruso[35], Silvia Colombo[36], Antonio Benedetti[37], Marco Marzioni[37], Andrea Galli[38], Fabio Marra[38], Mirko Tarocchi[38], Antonio Picciotto[39], Filomena Morisco[40], Luca Fabris[41], Lory Saveria Crocè[42], Claudio Tiribelli[42], Pierluigi Toniutto[43], Mario Strazzabosco[44]

[6] Azienda Ospedaliera Ospedali Riuniti Villa Sofia-Cervello, Palermo.
[7] Azienda Ospedaliera Universitaria Pisana, Pisa.
[8] Azienda Ospedaliera Valduce, Como.
[9] Azienda Ospedaliero-Universitaria di Parma, Parma.
[10] Department of Clinical Medicine, University of Bologna, Bologna.
[11] Department of Interdisciplinary Medicine, University Medical School, Bari.
[12] Department of Internal Medicine, AO Provincia di Lecco, Lecco.
[13] Department of Internal Medicine, Ospedale Fatebene Fratelli e Oftalmico, Milan.
[14] Department of Internal Medicine, San Pietro Hospital, Bergamo, Ponte San Pietro.
[15] Department of Medical and Surgical Sciences (DIMEC) University of Bologna, Bologna, Italy.
[16] Department of Medical and Surgical Sciences, Bologna University, Bologna.
[17] Department of Medico-Surgical Sciences and Biotechnologies, Polo Pontino, University Sapienza of Rome, Rome.
[18] Department of Medico-Surgical Sciences and Biotechnologies, Sapienza University of Rome, Viale dell'Università 37, 00185, Rome.

[19] Department of Surgery, Oncology and Gastroenterology, University of Padua, Padova.
[20] Department of Translational Medicine, Università del Piemonte Orientale UPO, 28100 Novara.
[21] Division of Gastroenterology & Hepatology, Center for Predictive Medicine, Gradenigo Hospital, Turin.
[22] Division of Gastroenterology and Hepatology, Fondazione IRCCS Ca' Granda Ospedale Maggiore Policlinico, Milan.
[23] Gastroenterologia Epatologia e Trapiantologia, Papa Giovanni XXIII Hospital, Bergamo.
[24] Gastroenterology & Hepatology Unit, Di.Bi.M.I.S., University of Palermo, Palermo.
[25] Gastroenterology Unit, Department Internal Medicine, Policlinico San Martino, University of Genoa, Genoa.
[26] Hepatology Unit, Department of Medical and Surgical Sciences, University Hospital of Bologna.
[27] Liver Center IRCCS San Raffaele Hospital, 20132 Milan, Italy..
[28] Department of Pathophysiology and Transplantation, Università degli Studi di Milano, Milan.
[27] Internal Medicine, Gastroenterology and Liver Unit, A. Gemelli Polyclinic, Sacro Cuore Catholic University, 20123 Rome.
[28] IRCCS Casa Sollievo della Sofferenza Hospital, San Giovanni Rotondo.
[29] Italian College of General Practicioners, ASL Bari.
[30] Magenta Hospital, Magenta.
[31] Medical Department, Desio Hospital, Desio.
[32] Ospedale Niguarda, Milan.
[33] San Paolo Hospital Medical School, Università di Milano, Milan.
[34] Santa Croce Carle Hospital, Cuneo.
[35] Sezione di Gastroenterologia e Epatologia, Dipartimento Biomedico di Medicina Interna e Specialistica (Di.Bi.M.I.S.) University of Palermo, Palermo.
[36] Treviglio Hospital, Treviglio.
[37] Università Politecnica delle Marche, Ancona.
[38] University of Florence, Florence.
[39] University of Genoa, Genoa.
[40] University of Naples, Federico II, Naples.
[41] University of Padova, Padova.
[42] University of Trieste, & Fondazione Italiana Fegato (FIF) Trieste.
[43] University of Udine, Udine.
[44] Yale University, New Haven, Connecticut 06511, USA.

SUPPLEMENTARY METHODS

Metrics Definitions

Sensitivity was defined as:

$$\frac{\text{True Positives}}{\text{True Positives } + \text{ False Negatives}}$$

Specificity was defined as:

$$\frac{\text{True Negatives}}{\text{True Negatives } + \text{ False Positives}}$$

Positive Predictive Value was defined as:

$$\frac{\text{True Positives}}{\text{True Positives } + \text{ False Positives}}$$

Negative Predictive Value was defined as:

$$\frac{\text{True Negatives}}{\text{True Negatives } + \text{ False Negatives}}$$

Accuracy was defined as:

$$\frac{\text{True Positives} + \text{True Negative}}{\text{True Positives} + \text{False Negative} + \text{False Positives} + \text{True Negative}} \text{ x } 100$$

The *Matthews correlation coefficient* was defined as:

$$\frac{\text{True Positives x True Negatives } - \text{ False Positives x False Negatives}}{\sqrt{(\text{True Positives } + \text{ False Positives})(\text{True Positives } + \text{ False Negatives})(\text{True Negatives } + \text{ False Positives})(\text{True Negatives } + \text{ False Negatives})}}$$

The *Youden's Index* was defined as:

$$\frac{\text{True Positives}}{\text{True Positives} + \text{False Negative}} + \frac{\text{True Negatives}}{\text{True Negatives} + \text{False Positives}} - 1$$

SUPPLEMENTARY TABLE LEGEND

Supplementary Table 1 Values of npresel and presel features that were evaluated in combination with other hyperparameters for model selection.
Abbreviations: npresel, number of pre-selected features; sel, number of selected features.

Supplementary Table 2 Univariate ranking of variants. Only the first 30 variants are shown as example due to space constraints.

Supplementary Table 3 Sets of hyperparameters evaluated before choosing the final model.
Abbreviations: corr, correlation value; npresel, number of pre-selected features; sel, number of selected features.

Supplementary Table 4. List of selected variants entering Logic Learning Machine (LLM) model.

Supplementary Table 5 Summary Table of performance based on different sets of hyperparameters.
“errmax” represents the maximum level of error for each rule included in the ruleset. In other words, this corresponded to the maximum percentage of cases belonging to output classes different from the predicted one which verified the rule.

Abbreviations: errmax, maximum level of error for each rule; PPV, Positive Predictive Value; NPV, Negative Predictive Value.

Supplementary Table 6. Covering and error of each condition within the best rule including sex.
“Covering” is the percentage of samples belonging to the class described by the rule, fulfilling that specific rule; “Error” is the percentage of samples belonging to the other classes fulfilling that specific rule.

Supplementary Table 7. Covering and error of each condition within the best rule without sex.
“Covering” is the percentage of samples belonging to the class described by the rule, fulfilling that specific rule; “Error” is the percentage of samples belonging to the other classes fulfilling that specific rule.

SUPPLEMENTARY TABLES

Supplementary Table S1

	**Npresel**	**Sel**					
**1**	200	50	98				
**2**	248	62	110				
**3**	296	74	122				
**4**	344	86	134				
**5**	392	98	146	194			
**6**	440	110	158	206			
**7**	488	122	170	218			
**8**	536	134	182	230			
**9**	584	146	194	242	290		
**10**	632	158	206	254	302		
**11**	680	170	218	266	314		
**12**	728	182	230	278	326		
**13**	776	194	242	290	338	386	
**14**	824	206	254	302	350	398	
**15**	872	218	266	314	362	410	
**16**	920	230	278	326	374	422	
**17**	968	242	290	338	386	434	482
**18**	1016	254	302	350	398	446	494

Supplementary Table S2

Attribute	Score	rank
Sex	0.187156	1
6:32653792:A:G	0.034963	2
3:119262734:C:CT	0.018558	3
3:119111870:C:T	0.016562	4
7:37176353:C:CA	0.016303	5
7:128588434:T:TG	0.015746	6
7:128589000:C:T	0.015746	7
3:119103580:G:T	0.015431	8
3:119116150:A:G	0.015268	9
7:37176353:C:CA	0.015237	10
7:128714746:A:G	0.015067	11
7:128714843:C:T	0.015067	12
7:128715299:A:T	0.015067	13
17:43919070:C:T	0.015019	14
17:43919068:G:T	0.015019	15
17:43919073:G:T	0.015019	16
19:50927358:A:G	0.01479	17
7:128717234:A:AAT	0.014777	18
7:128717305:A:G	0.014777	19
3:119128398:A:G	0.014731	20
3:119130141:A:G	0.014731	21
4:103446115:A:G	0.014575	22
16:11082692:C:T	0.014553	23
16:11058753:A:C	0.014541	24
7:128713630:A:G	0.014506	25
11:64031798:C:G	0.014402	26
11:64031798:C:G	0.014381	27
16:11195948:A:G	0.014345	28
7:128716007:G:T	0.014325	29
1:67875102:A:ACC	0.01418	30

Supplementary Table S3

Run number	Corr	Split	Npresel	Nsel
1	0.8	10	1016	254
2	0.8	10	1016	302
3	0.8	10	1016	350
4	0.8	10	1016	398
5	0.8	10	1016	446
6	0.8	10	1016	494
7	0.8	10	968	242
8	0.8	10	968	290
9	0.8	10	968	338
10	0.8	10	968	386
11	0.8	10	968	434
12	0.8	10	968	482
13	0.8	10	920	230
14	0.8	10	920	278
15	0.8	10	920	326
16	0.8	10	920	374
17	0.8	10	920	422
18	0.8	10	872	218
19	0.8	10	872	266
20	0.8	10	872	314
21	0.8	10	872	362
22	0.8	10	872	410
23	0.8	10	824	206
24	0.8	10	824	254
25	0.8	10	824	302
26	0.8	10	824	350
27	0.8	10	824	398
28	0.8	10	776	194
29	0.8	10	776	242
30	0.8	10	776	290
31	0.8	10	776	338
32	0.8	10	776	386
33	0.8	10	728	182
34	0.8	10	728	230
35	0.8	10	728	278
36	0.8	10	728	326
37	0.8	10	680	170
38	0.8	10	680	218
39	0.8	10	680	266

40	0.8	10	680	314
41	0.8	10	632	158
42	0.8	10	632	206
43	0.8	10	632	254
44	0.8	10	632	302
45	0.8	10	584	146
46	0.8	10	584	194
47	0.8	10	584	242
48	0.8	10	584	290
49	0.8	10	536	134
50	0.8	10	536	182
51	0.8	10	536	230
52	0.8	10	488	122
53	0.8	10	488	170
54	0.8	10	488	218
55	0.8	10	440	110
56	0.8	10	440	158
57	0.8	10	440	206
58	0.8	10	392	98
59	0.8	10	392	146
60	0.8	10	392	194
61	0.8	10	344	86
62	0.8	10	344	134
63	0.8	10	296	74
64	0.8	10	296	122
65	0.8	10	248	62
66	0.8	10	248	110
67	0.8	10	200	50
68	0.8	10	200	98
69	0.7	10	1016	254
70	0.7	10	1016	302
71	0.7	10	1016	350
72	0.7	10	1016	398
73	0.7	10	1016	446
74	0.7	10	1016	494
75	0.7	10	968	242
76	0.7	10	968	290
77	0.7	10	968	338
78	0.7	10	968	386
79	0.7	10	968	434
80	0.7	10	968	482
81	0.7	10	920	230
82	0.7	10	920	278

83	0.7	10	920	326
84	0.7	10	920	374
85	0.7	10	920	422
86	0.7	10	872	218
87	0.7	10	872	266
88	0.7	10	872	314
89	0.7	10	872	362
90	0.7	10	872	410
91	0.7	10	824	206
92	0.7	10	824	254
93	0.7	10	824	302
94	0.7	10	824	350
95	0.7	10	824	398
96	0.7	10	776	194
97	0.7	10	776	242
98	0.7	10	776	290
99	0.7	10	776	338
100	0.7	10	776	386
101	0.7	10	728	182
102	0.7	10	728	230
103	0.7	10	728	278
104	0.7	10	728	326
105	0.7	10	680	170
106	0.7	10	680	218
107	0.7	10	680	266
108	0.7	10	680	314
109	0.7	10	632	158
110	0.7	10	632	206
111	0.7	10	632	254
112	0.7	10	632	302
113	0.7	10	584	146
114	0.7	10	584	194
115	0.7	10	584	242
116	0.7	10	584	290
117	0.7	10	536	134
118	0.7	10	536	182
119	0.7	10	536	230
120	0.7	10	488	122
121	0.7	10	488	170
122	0.7	10	488	218
123	0.7	10	440	110
124	0.7	10	440	158
125	0.7	10	440	206

126	0.7	10	392	98
127	0.7	10	392	146
128	0.7	10	392	194
129	0.7	10	344	86
130	0.7	10	344	134
131	0.7	10	296	74
132	0.7	10	296	122
133	0.7	10	248	62
134	0.7	10	248	110
135	0.7	10	200	50
136	0.7	10	200	98
137	0.9	10	1016	254
138	0.9	10	1016	302
139	0.9	10	1016	350
140	0.9	10	1016	398
141	0.9	10	1016	446
142	0.9	10	1016	494
143	0.9	10	968	242
144	0.9	10	968	290
145	0.9	10	968	338
146	0.9	10	968	386
147	0.9	10	968	434
148	0.9	10	968	482
149	0.9	10	920	230
150	0.9	10	920	278
151	0.9	10	920	326
152	0.9	10	920	374
153	0.9	10	920	422
154	0.9	10	872	218
155	0.9	10	872	266
156	0.9	10	872	314
157	0.9	10	872	362
158	0.9	10	872	410
159	0.9	10	824	206
160	0.9	10	824	254
161	0.9	10	824	302
162	0.9	10	824	350
163	0.9	10	824	398
164	0.9	10	776	194
165	0.9	10	776	242
166	0.9	10	776	290
167	0.9	10	776	338
168	0.9	10	776	386

169	0.9	10	728	182
170	0.9	10	728	230
171	0.9	10	728	278
172	0.9	10	728	326
173	0.9	10	680	170
174	0.9	10	680	218
175	0.9	10	680	266
176	0.9	10	680	314
177	0.9	10	632	158
178	0.9	10	632	206
179	0.9	10	632	254
180	0.9	10	632	302
181	0.9	10	584	146
182	0.9	10	584	194
183	0.9	10	584	242
184	0.9	10	584	290
185	0.9	10	536	134
186	0.9	10	536	182
187	0.9	10	536	230
188	0.9	10	488	122
189	0.9	10	488	170
190	0.9	10	488	218
191	0.9	10	440	110
192	0.9	10	440	158
193	0.9	10	440	206
194	0.9	10	392	98
195	0.9	10	392	146
196	0.9	10	392	194
197	0.9	10	344	86
198	0.9	10	344	134
199	0.9	10	296	74
200	0.9	10	296	122
201	0.9	10	248	62
202	0.9	10	248	110
203	0.9	10	200	50
204	0.9	10	200	98

Supplementary Table S4

6:32653792:A:G
Sex
7:128588434_1
7:128590801_1
7:128669912_1
7:128681062_1
7:128705730_1
7:128705730_2
7:128714746_1
7:128718178_1
7:128720295_1
7:128723194_1
7:128723943_1
10:49999680_1
10:50003921_1
11:63908660_2
11:64011614_2
11:64011854_2
11:64017417_1
11:64021605_1
11:64031798_1
11:64031798_2
11:64053157_1
11:64102948_1
11:64158950_2
12:6212681_1
12:6495275_2
13:42969049_1
13:42970446_1
13:42970446_2
14:68976059_1
14:68976059_2
14:92928693_2
14:92932650_2

14:103563195_2
14:103563421_2
14:103563547_2
16:11033402_1
16:11035888_1
16:11045340_1
16:11058753_1
16:11058757_1
16:11082692_1
16:11085646_1
16:11090358_1
16:11189256_1
16:11191219_1
16:11193553_1
16:11195948_1
16:11221584_1
16:11233179_1
16:11239689_1
16:27165173_1
16:86073712_1
16:86076497_1
16:86079249_1
16:86079388_1
16:86212152_1
16:86213219_1
16:86214690_1
17:37952989_1
17:38020058_2
17:38023745_2
17:38024626_2
17:38026169_2
17:38031030_2
17:38031714_2
17:38031857_2

17:38038389_2
17:38044893_2
17:38045725_2
17:38049589_2
17:38055921_1
17:38055921_2
17:38057189_1
17:38057189_2
17:38066267_2
17:38067533_2
17:38070071_2
17:43899417_1
17:43900215_1
17:43902861_1
17:43903298_1
17:43906828_1
17:43908989_1
17:43910183_1
17:43912635_1
17:43919070_1
17:43919096_1
17:43925966_1
17:43928614_1
17:43932129_1
17:43932173_1
17:43933190_1
17:43933579_1
17:43933673_1
17:43945745_1
17:43945938_1
17:43946223_1
17:43946318_1
17:43946423_1
17:43946875_1

17:43948977_1
17:43949342_1
17:43949448_1
17:43952944_1
17:43954416_1
17:43956139_1
17:43958362_1
17:43960341_1
17:43977049_1
17:43978534_1
17:43991515_1
17:43994252_1
17:43994648_1
17:43996430_1
17:44003397_1
17:44006453_1
17:44010452_1
17:44010463_1
17:44012463_1
17:44017666_1
17:44018399_1
17:44018488_1
17:44019107_1
17:44023087_1
17:44023828_1
17:44025359_1
17:44033097_1
17:44036408_1
17:44038536_1
17:44038785_1
17:44039008_1
17:44040288_1
17:44040823_1
17:44043092_1

17:44047449_1
17:44061608_1
17:44065263_1
17:44078816_1
17:44080465_1
17:44081064_1
17:44089727_1
17:44094471_1
17:44096553_1
17:44103825_1
17:44125288_1
17:44126575_1
17:44133818_1
17:44137009_1
17:44137386_1
17:44147574_1
17:44147721_1
17:44150161_1
17:44156180_1
17:44157676_1
17:44163547_1
17:44181933_1
17:44183317_1
17:44184819_1
17:44185431_1
17:44188755_1
17:44192590_1
17:44196447_1
17:44200015_1
17:44201109_1
17:44205690_1
17:44207887_1
17:44222335_1
17:44228529_1

17:44257473_1
17:44258422_1
17:44272679_1
17:44283022_1
19:10645576_1
19:18236725_1
19:18244560_1
19:50924093_1
19:50925395_1
19:50926265_1
19:50926742_1
19:50927358_1
2:25332696_2
2:135188248_1
2:191917317_1
2:191943742_1
2:191992237_1
2:191992611_1
3:17131082_2
3:119103580_1
3:119111870_1
3:119116150_1
3:119128398_1
3:119174383_1
3:119209027_1
3:119219934_1
3:119222456_1
3:119228508_1
3:119244593_1
1:67875102_1
3:119257802_1
3:119262734_1
3:119262734_2
3:119286713_1

3:119292618_1
3:119297389_1
3:159497430_1
3:159520115_1
3:159556462_1
3:159561337_1
3:159568546_1
3:159726324_1
4:103444533_1
4:103446115_1
4:103476166_1
4:103511747_1
4:103531112_1
4:103538911_1
4:103540780_1
4:103554350_1
4:103555611_1
4:103559876_1
4:103622568_1
4:103622568_2
7:37176353_1
7:37176353_2

Supplementary Table S5

**Configuration**	1
**set**	test
**errmax**	0,05
**Accuracy**	71,670276
**Matthews**	0,289932
**Youden**	0,21597
**Sensitivity**	0,288288
**Specificity**	0,927858
**PPV**	0,663212
**NPV**	0,725694
**Positive_LR**	3,996119
**Negative_LR**	0,767048
**Hyperparameter_Set**	0.8_872_266_10

Supplementary Table S6

		Covering of the condition	Error of the condition
**Condition 1**	14:92932650_2 = "C"	0.90	0.11
**Condition 2**	17:43906828_1 = "G"	0.00	0.11
**Condition 3**	17:43912635_1 = "A"	0.00	0.11
**Condition 4**	17:44038536_1 = "CA"	0.45	0.22
**Condition 5**	17:44040823_1 = "C"	0.00	0.11
**Condition 6**	17:44065263_1 = "T"	11.49	5.44
**Condition 7**	17:44183317_1 = "C"	0.45	0.11
**Condition 8**	17:44185431_1 = "T"	0.00	0.11
**Condition 9**	17:44222335_1 = "G"	0.00	0.11
**Condition 10**	17:44283022_1 = "A"	0.00	0.11
**Condition 11**	3:119111870_1 = "T"	4.95	3.66
**Condition 12**	7:128705730_1 = "T"	1.58	0.44
**Condition 13**	Sex = F	0.90	5.88

Supplementary Table S7

		Covering of the condition	Error of the condition
**Condition 1**	17:38020058_2 = "AC"	0.00	0.11
**Condition 2**	17:38049589_2 = "T"	0.45	0.55
**Condition 3**	17:38070071_2 = "C"	0.23	0.11
**Condition 4**	17:43933579_1 = "C"	7.88	6.33
**Condition 5**	2:135188248_1 = "A"	1.13	0.67
**Condition 6**	2:25332696_2 = "C"	1.80	1.33
**Condition 7**	3:159726324_1 = "A"	0.45	1.11

SUPPLEMENTARY FIGURE LEGEND

Supplementary Figure S1 Calibration plot with Brier score.

SUPPLEMENTARY FIGURES

Supplementary Figure S1

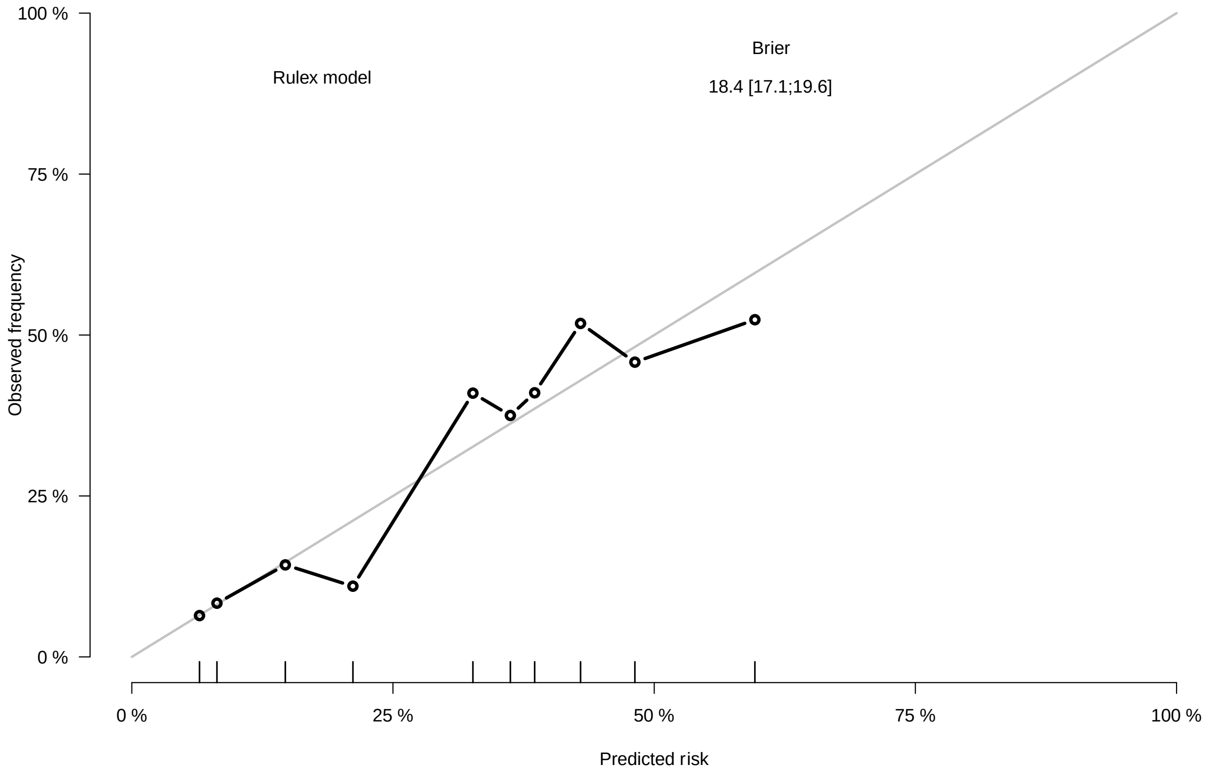

Supplement: Supplementary file 1 [file jpm-12-01587-s001.zip › SUPPLEMENTARY MATERIAL.pdf]
